# Supplementary material for: Momentum-independent magnetic excitation continuum in the honeycomb iridate H3LiIr2O6
Source: Nat Commun. 2023 Aug 18;14:5018. doi: 10.1038/s41467-023-40769-x (PMC10439105; doi:10.1038/s41467-023-40769-x)
Supplement: Supplementary file 1 — Supplementary Information [file 41467_2023_40769_MOESM1_ESM.pdf]

Supplementary Information for Momentum-independent magnetic excitation continuum in the honeycomb iridate $H_3LiIr_2O_6$

A. de la Torre,[1] B. Zager,[1] F. Bahrami,[2] M. H. Upton,[3] J. Kim,[3] G. Fabbris,[3] G.-H. Lee,[4] W. Yang,[4] D. Haskel,[3] F. Tafti,[2] and K. W. Plumb[1]

[1]*Department of Physics, Brown University, Providence, Rhode Island 02912, United States*
[2]*Department of Physics, Boston College, Chestnut Hill, MA 02467, USA*
[3]*Advanced Photon Source, Argonne National Laboratory, Argonne, Illinois 60439, USA*
[4]*Advanced Light Source, Lawrence Berkeley National Laboratory, Berkeley 94720, USA*
(Dated: July 18, 2023)

CONTENTS

Supplementary Note 1, Incident energy of the RIXS spectra 2

Supplementary Note 2, Details of the crystal field analysis 2

Supplementary Note 3, Momentum dependence of crystal field excitations 3

Supplementary Note 4, X-ray Absorption Spectroscopy measurements in $H_3LiIr_2O_6$ 4

Supplementary Note 5, Polarization dependence of the RIXS intensity at the α-Li_2IrO_3 magnetic wavector 5

Supplementary References 5

SUPPLEMENTARY NOTE 1, INCIDENT ENERGY OF THE RIXS SPECTRA

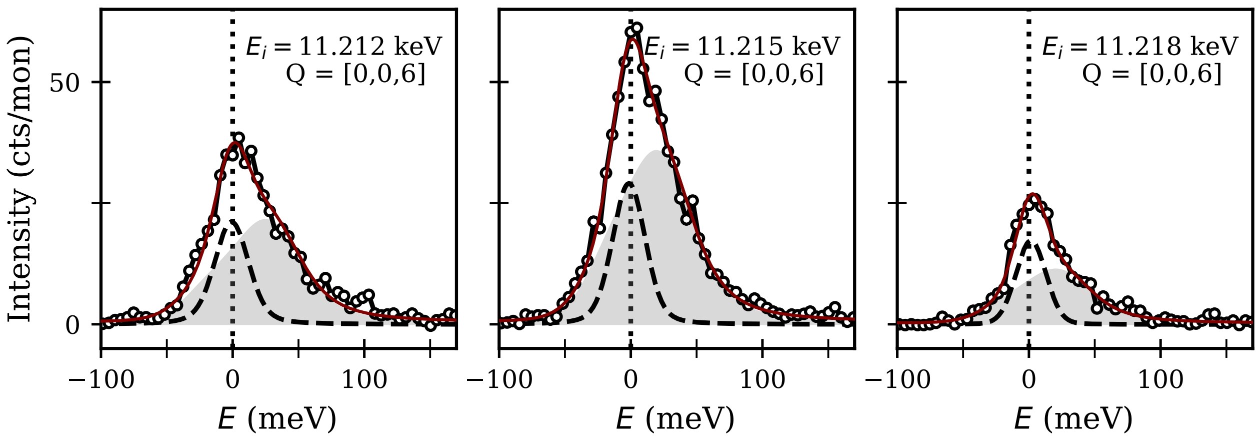

Supplementary Figure 1. Room temperature resonant inelastic X-ray spectra of $H_3LiIr_2O_6$ at $Q = [0, 0, 6]$ at three different values of E_i showing the resonant behavior of the magnetic excitation continuum. Energy resolution for this data set was set to FWHM = 28 meV. Maroon solid line is a fit to a Voigt profile for the elastic intensity (dashed line) and an overdamped harmonic oscillator for the inelastic intensity (grey shading).

In Supplementary Figure 1, we show room temperature RIXS spectra of $H_3LiIr_2O_6$ at $Q = [0, 0, 6]$ at three different X-ray incident energies. The continuum of magnetic excitations displays resonant behavior at the Ir L_3 edge, as signified by the change of intensity of the overdamped harmonic oscillator (grey solid area) with incident x-ray energy. As discussed in Ref. [1] this resonant behavior is consistent with a magnetic spin-flip excitation. Phonon excitations require a more delocalized intermidiate state and are expected to resonate at the enhanced e_g resonance. This fact in combination with a scattering geometry with $2\theta = 90°$ further supports a magnetic origin of the low energy excitations in $H_3LiIr_2O_6$ .The extracted center energy, $E = 38 \pm 5$ meV, and width, $\sigma = 45 \pm 5$ meV are consistent with those found with the higher resolution data shown in Fig. 1 of the main text.

SUPPLEMENTARY NOTE 2, DETAILS OF THE CRYSTAL FIELD ANALYSIS

In Supplementary Figure 2 **a** we reproduce the data from Fig. 4 **b** highlighting the four Gaussians peaks (A -D) needed to account for the intra-t_{2g} excitations centered at $E_A = 504.32$ meV, $E_B = 639.38$ meV, $E_C = 787.89$ and $E_D = 919.48$ meV. In Supplementary Figure 2 **b** we show RIXS data for α-Li_2IrO_3 digitized from Ref. [2]. The grey shaded curve shows the calculated RIXS intensity from the exact diagonalization of a Hamiltonian in the large cubic crystal field limit $H = H_U + H_{CF} + H_t$ including spin orbit coupling ($\lambda = 540$ meV), on-site Coulomb interactions (H_U), trigonal distortions H_{CF} and nearest-neighbors hopping H_t [3, 4]. To account for local disorder induced broadening, the trigonal fields δ, and leading hopping integrals t_O and $t_{||}$ are randomly sampled from a normal distribution with standard deviation $\sigma_{t_0} = 50$ meV and $\sigma_{t_{||}} = 10$ meV. The standard deviation for δ is allowed to vary between $H_3LiIr_2O_6$, $\sigma_\delta = 20$ meV, and α-Li_2IrO_3 $\sigma_\delta = 5$ meV. Our calculations explore the phase diagram given by the mean values (δ,t_O,$t_{||}$) to find the set of parameters that better account for the observed RIXS intensity. For $H_3LiIr_2O_6$ we find $\delta = -47$ meV, $t_O = 440$ meV and $t_{||} = -50$ meV and for α-Li_2IrO_3 $\delta = -50$ meV, $t_O = 400$ meV and $t_{||} = -30$ meV.

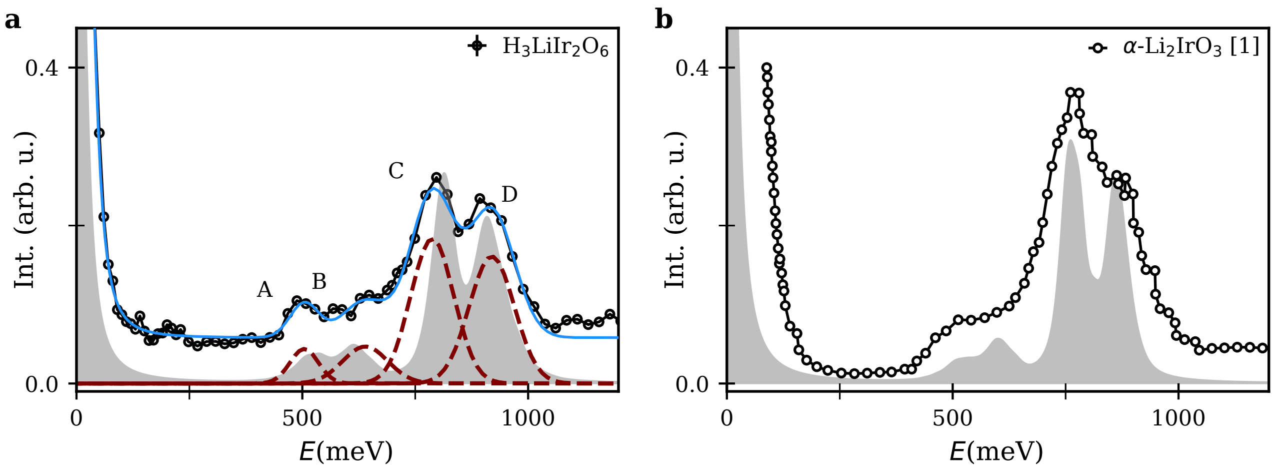

Supplementary Figure 2. **a** Intra-t_{2g} RIXS excitations at $E_i = 11.215$ keV (circular markers) compared to the calculated RIXS intensity from exact diagonalization calculations (grey shading). Blue solid line is a fit to the data including a Voigt peak, a DHO, four Gaussian peaks (red dashed lines) and an arctan step to account for the background. **b** Intra-t_{2g} RIXS excitations at $E_i = 11.215$ keV (circular markers) extracted from [2] compared to the calculated RIXS intensity (grey shading).

SUPPLEMENTARY NOTE 3, MOMENTUM DEPENDENCE OF CRYSTAL FIELD EXCITATIONS

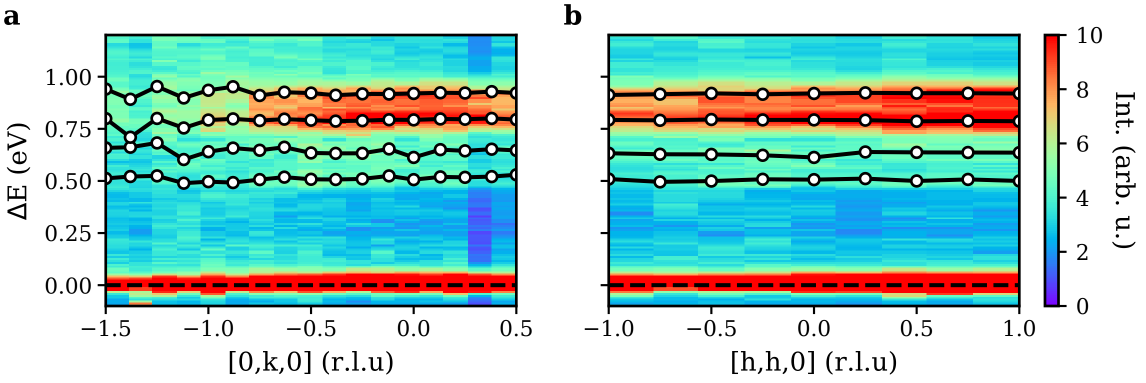

Supplementary Figure 3. Momentum dependence of the RIXS intensity of the intra-t_{2g} crystal field excitations along **a** $[0, k, 0]$ and **b** $[h, h, 0]$. Circular markers indicate the extract energy from a fit to the data as described in the previous section.

Supplementary Figure 3 **a** and **b** shows the intensity variation of the high-energy RIXS spectrum as a function of momentum along $[0, k, 0]$ and $[h, h, 0]$ respectively. We observed differences of up to a factor of 2 in the inelastic intensity at equivalent momentum points but no dispersion of the crystal field (CF) excitations was observed. The CFs intensity variation is not intrinsic to $H_3LiIr_2O_6$. To collect the momentum dependent data shown in Fig. 1, 2 and 3 of the main text and in Supplementary Figure 3, we vary θ, the X-ray incidence angle, ϕ, the azimuthal angle, and χ, defining the tilt of the sample normal with respect to the scattering plane. Given the sample size (40μm $\times$ 40μm) comparable to the spot size of the X-ray beam, we cannot exclude that the observed intensity variation is related to a small walk of the sample away from the center of rotation. Another possible extrinsic artifact at play are self-absorption effects dependent on the values of θ and 2θ, the scattering angle, and on the incident and outgoing energy which are known to occur near resonance in RIXS experiments at the Ir L_3 edge [1].

SUPPLEMENTARY NOTE 4, X-RAY ABSORPTION SPECTROSCOPY MEASUREMENTS IN $H_3LIIR_2O_6$

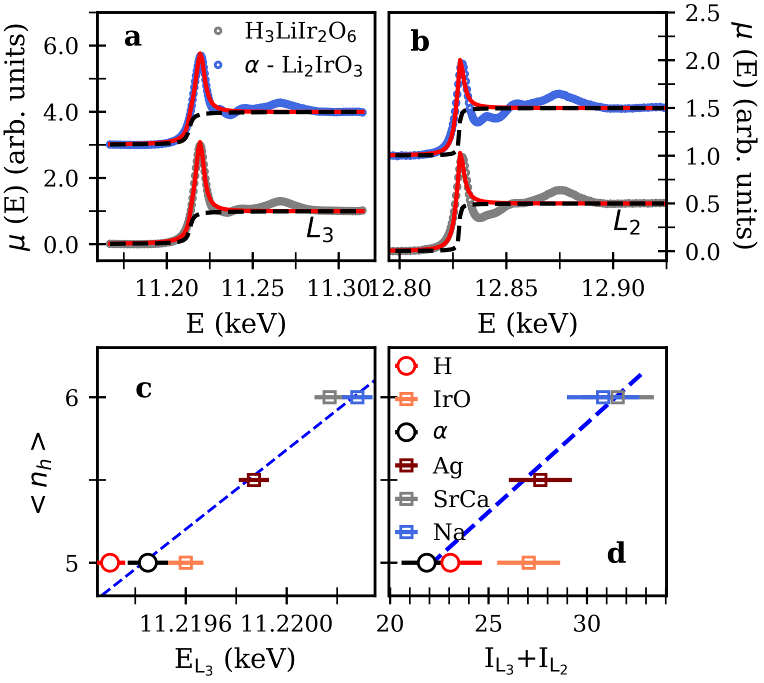

Supplementary Figure 4. **a** Ir L_3 edge XAS intensity in $H_3LiIr_2O_6$ (blue markers) and α-Li_2IrO_3 (grey markers). **b** Ir L_2 edge XAS intensity in $H_3LiIr_2O_6$ (blue markers) and α-Li_2IrO_3 (grey markers). Red line is a fit to a lo Lorentzian peak and an arctangent step (dotted black line). All data was taken at $T = 300$ K. **c** Direct comparison of the L_3 white line intensity of $H_3LiIr_2O_6$ (H) to that of α-Li_2IrO_3 (α) and standard compounds $Sr_3CaIr_2O_9$ (SrCa), $NaIrO_3$ (Na), $Ag_3LiIr_2O_6$ (Ag) and IrO. **d** Same as **c** but showing the integrated intensity $L_3 + L_2$.

In Supplementary Figure 4 **a** and**b**, we show the XAS intensity of $H_3LiIr_2O_6$ and α-Li_2IrO_3 at the Ir L_3 and L_2 edge, respectively. No change is observed in either the L_3 white line position or $L_3 + L_2$ integrated intensity shown in Supplementary Figure 4**c** and**d**. Thus, the introduction of H does not modify the Ir^{4+} oxidation state in $H_3LiIr_2O_6$.

SUPPLEMENTARY NOTE 5, POLARIZATION DEPENDENCE OF THE RIXS INTENSITY AT THE α-LI_2IRO_3 MAGNETIC WAVECTOR

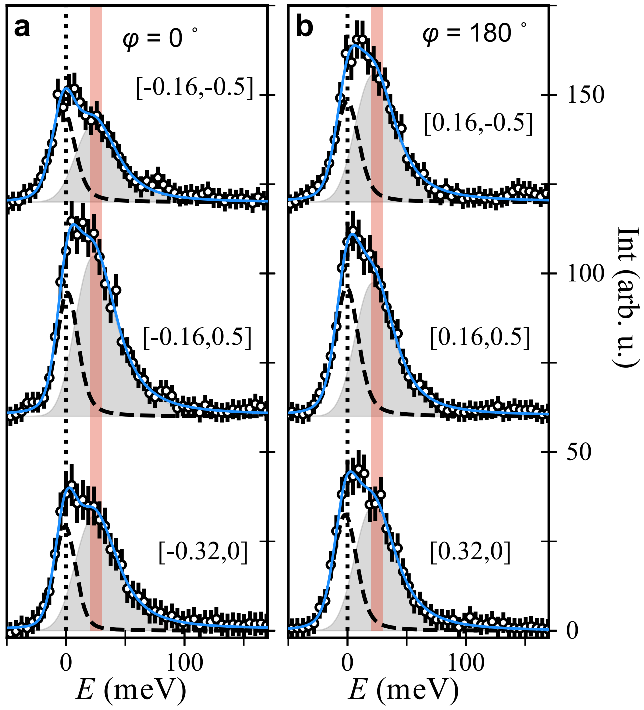

Supplementary Figure 5. **a** $T = 10$ K RIXS intensity at the wavevectors of the 120° spiral order of α-Li_2IrO_3 for $\varphi = 0°$ and, **b**, $\varphi = 180°$. Blue solid line is a fit to the data including a Voigt profile for the elastic line (dotted black line) and a damped harmonic oscillator (grey shading) centered at $E_0 = 25$ meV (red bar of width 10 meV)

In Supplementary Figure 5 **a** and **b** we compare the RIXS spectra at $q_\alpha = [\pm 0.16, -0.5], [\pm 0.16, 0.5], [\pm 0.32, 0]$, the ordering vectors for the three 120° domains of the incommensurate spiral order in α-Li_2IrO_3 [5] at two different azimuthal angles $\varphi = 0, 180°$. The RIXS spectra can be fitted with the same functional form.

SUPPLEMENTARY REFERENCES

[1] A. Revelli, M. Moretti Sala, G. Monaco, C. Hickey, P. Becker, F. Freund, A. Jesche, P. Gegenwart, T. Eschmann, F. L. Buessen, S. Trebst, P. H. M. van Loosdrecht, J. van den Brink, and M. Grüninger, "Fingerprints of Kitaev physics in the magnetic excitations of honeycomb iridates," Physical Review Research **2**, 043094 (2020).

[2] H. Gretarsson, J. P. Clancy, X. Liu, J. P. Hill, Emil Bozin, Yogesh Singh, S. Manni, P. Gegenwart, Jungho Kim, A. H. Said, D. Casa, T. Gog, M. H. Upton, Heung-Sik Kim, J. Yu, Vamshi M. Katukuri, L. Hozoi, Jeroen van den Brink, and Young-June Kim, "Crystal-Field Splitting and Correlation Effect on the Electronic Structure of A_2IrO_3," Phys. Rev. Lett. **110**, 076402 (2013).

[3] A. de la Torre, B. Zager, F. Bahrami, M. DiScala, J. R. Chamorro, M. H. Upton, G. Fabbris, D. Haskel, D. Casa, T. M. McQueen, F. Tafti, and K. W. Plumb, "Enhanced hybridization in the electronic ground state of the intercalated honeycomb iridate $Ag_3LiIr_2O_6$," Phys. Rev. B **104**, L100416 (2021).

[4] A. de la Torre, B. Zager, J. R. Chamorro, M. H. Upton, G. Fabbris, D. Haskel, D. Casa, T. M. McQueen, and K. W. Plumb, "Electronic ground state of two nonmagnetic pentavalent honeycomb iridates," Phys. Rev. Materials **6**, 084406 (2022).

[5] Sae Hwan Chun, P. Peter Stavropoulos, Hae-Young Kee, M. Moretti Sala, Jungho Kim, Jong-Woo Kim, B. J. Kim, J. F. Mitchell, and Young-June Kim, "Optical magnons with dominant bond-directional exchange interactions in the honeycomb lattice iridate $\alpha-Li_2IrO_3$," Physical Review B **103**, L020410 (2021).
